# Gendering Neighbourhood Marginalization Metrics in Mental Health Services Research: A Cross-Sectional Exploration of a Rural and Small Urban Population

**DOI:** 10.3390/ijerph182111197

**Published:** 2021-10-25

**Authors:** Neeru Gupta, Dan Lawson Crouse, Ismael Foroughi, Thalia Nikolaidou

**Affiliations:** 1 Department of Sociology, University of New Brunswick, PO Box 4400, Fredericton, NB E3B 5A3, Canada; i.foroughi@unb.ca; 2 Health Effects Institute, Boston, MA 02110, USA; DCrouse@healtheffects.org; 3 Department of Geodesy and Geomatics Engineering, University of New Brunswick, PO Box 4400, Fredericton, NB E3B 5A3, Canada; Thalia.Nikolaidou@unb.ca

**Keywords:** socioenvironmental determinants of health, mental health services research, gender and health, population health surveillance, residence characteristics, spatial epidemiology, data linkage

## Abstract

***Background:*** Little is known about the extent to which socioenvironmental characteristics may influence mental health outcomes in smaller population centres or differently among women and men. This study used a gender-based analysis approach to explore individual- and neighbourhood-level sex differences in mental health service use in a context of uniquely smaller urban and rural settlements. ***Methods:*** This cross-sectional analysis leveraged multiple person-based administrative health datasets linked with geospatial datasets among the population aged 1 and over in the province of New Brunswick, Canada. We used multinomial logistic regression to examine associations between neighbourhood characteristics with risk of service contacts for mood and anxiety disorders in 2015/2016, characterizing the areal measures among all residents (gender neutral) and by males and females separately (gender specific), and controlling for age group. ***Results:*** Among the province’s 707,575 eligible residents, 10.7% (females: 14.0%; males: 7.3%) used mental health services in the year of observation. In models adjusted for gender-neutral neighbourhood characteristics, service contacts were significantly more likely among persons residing in the most materially deprived areas compared with the least (OR = 1.09 [95% CI: 1.05–1.12]); when stratified by individuals’ sex, the risk pattern held for females (OR = 1.13 [95% CI: 1.09–1.17]) but not males (OR = 1.00 [95% CI: 0.96–1.05]). Residence in the most female-specific materially deprived neighbourhoods was independently associated with higher risk of mental health service use among individual females (OR = 1.08 [95% CI: 1.02–1.14]) but not among males (OR = 1.02 [95% CI: 0.95–1.10]). ***Conclusion:*** These findings emphasize that research needs to better integrate sex and gender in contextual measures aiming to inform community interventions and neighbourhood designs, notably in small urban and rural settings, to reduce socioeconomic inequalities in the burden of mental disorders.

## 1. Introduction

It has been widely postulated that more egalitarian social and residential environments can level the health of populations [1,2]. A growing body of empirical evidence suggests that selected characteristics of neighbourhood environments—often based on summary indices of multiple deprivation from population census data—are related to higher morbidity and healthcare utilization, even in contexts of universal healthcare coverage such as Canada [3,4,5,6]. Health systems can play an important role in addressing such disparities through policies and programs targeting improved equity in physical and mental health outcomes [7]. Much of the research linking health and neighbourhood characteristics, however, focuses on physical endpoints, such as cardiometabolic, respiratory, or maternal-perinatal health outcomes [8,9]. Some studies have examined associations between neighbourhoods and mental health outcomes; however, they have tended to be limited to large, urban contexts [4,10,11,12,13] or to specific patient groups [5,14,15]. Little is known about the influence of neighbourhood environments on mental health service contacts among the general population in smaller urban and rural settlements. Even less is known about the role of sex-specific, or gender-based, indicators of inequality at the neighbourhood level on such outcomes [16,17]. In other words, despite the recognized importance of considering sex and gender in health services research and practice, structures in inequality that may shape gendered differences in health remain underexamined in the literature [18,19].

A greater propensity for women to experience common mental disorders and to seek services for mental health issues has been widely observed in Canada and elsewhere [12,20,21], underscoring the need for research on the psychosocial factors perpetuating gender differences in mental health [22]. Yet, there is a dearth of evidence on whether gender-neutral population health metrics, that is, study designs that do not explicitly integrate sex or gender variables from the onset, may yield findings which may ultimately help perpetuate and exacerbate disparities in mental health outcomes for women and men. Moreover, while interest is growing in examining how individuals’ health and healthcare experiences may differ by the characteristics of their residential environment, and how deprivation measures may be optimized in supporting regionally relevant interpretations, there is a surprising lack of consensus in which neighbourhood characteristics are most salient [17,23,24,25].

The measurements of neighbourhood-level sources of chronic stressors related to gender inequality and to rurality are relatively understudied for informing community health improvement [13,16]. The objective of this population-based study was to investigate correlations between sex-specific neighbourhood socioeconomic characteristics and mental health service use in New Brunswick, a Canadian province of smaller urban and rural areas. Drawing on multiple linked administrative health and geospatial datasets, we aimed to address the following research questions: (i) are there sex-specific associations between neighbourhood characteristics with individuals’ risk of service contacts for mood and anxiety disorders; and (ii) do these associations remain after controlling for sex-disaggregated neighbourhood-level characteristics. Considering gender-based analysis as a process to interrogate relational issues between men and women rather than a concrete measure [26], we explored and operationalized different alternatives of area deprivation measures reflecting male- vs. female-specific neighbourhood gender inequality. We hypothesized that women residing in neighbourhoods of higher socioeconomic marginalization, and especially in those of higher female-specific marginalization, would have higher risks of mental health service contacts relative to men and relative to women residing in neighbourhoods of male-specific marginalization.

## 2. Materials and Methods

### 2.1. Setting

One of Canada’s less populous provinces, New Brunswick accounts for 2.1% of the national population and is characterized by uniquely rural and small urban communities: 51.0% reside in rural areas (compared with 18.7% nationally), and the three main urban areas—Moncton, Saint John, and Fredericton—each have a population under 150,000 [27]. As in the other provinces and territories, all New Brunswick residents are eligible for publicly financed insurance for essential medical and hospital services, a universal coverage system known as Medicare [28]. Access points to mental health services include hospitals, physician offices, community-based clinics, schools, mobile counselling services, and justice settings [29]. Survey data indicate that 11.5% (95% CI: 9.1–14.4%) of New Brunswickers perceived their mental health to be poor or fair, a rate statistically similar to the national average [30]. Most New Brunswickers report having a regular healthcare provider (90.6%), and this at a rate significantly higher than the national average (85.8%) [30]. There is some evidence that, among persons living with selected chronic conditions, the risk of service use for mental health disorders is higher among those living in materially deprived neighbourhoods [3,31].

### 2.2. Study Design and Participants

We used multiple linkable provincial administrative and geospatial socioenvironmental datasets for this population-based cross-sectional study. Firstly, we leveraged pseudonymized administrative health datasets on: (i) New Brunswick Medicare registrations, which captured individual-level information on sex (male/female binary), age, and residential history [32]; and (ii) case ascertainments of medical and hospital service contacts for mood and anxiety disorders by fiscal year [33]. We used the latest health services surveillance data available at the time of analysis, that is, relating to the 2015/2016 fiscal year. Thanks to the universal coverage system, the data are considered virtually complete: fewer than 3% of the total population are excluded from the provincial administrative datasets, owing to coverage of certain groups under federal health programs (e.g., those serving in the military, inmates of federal correctional institutions, some refugee protection claimants). Secondly, the administrative datasets were linked deterministically by residential postal code to new and existing census-based geocoded datasets of neighbourhood socioeconomic indicators. We excluded from the study population those not having reached their first birthday in the year of observation and those without valid postal code information.

### 2.3. Health Service Contacts for Mood and Anxiety Disorders

The outcome of interest was having any service contact in the year for mood and anxiety disorders, the most common mental disorders and among the most important contributors to disease burden in Canada [34]. Case ascertainments of service use for a mood disorder (e.g., depression, dysthymia, bipolar disorder) and/or an anxiety disorder (e.g., social phobia, post-traumatic stress disorder, obsessive-compulsive disorder) were based on a validated algorithm tracing individuals’ interactions with the publicly-insured healthcare system among the population aged 1 year or over [34,35]. Cases of service contacts were defined in accordance with the Canadian Chronic Diseases Surveillance System (CCDSS), based on recorded diagnoses and nomenclature of meeting screening criteria mapped to the International Classification of Diseases, 10th revision (ICD-10 codes F30–F42; F44–F48; F68) [33]. The CCDSS criteria were validated elsewhere as strongly able to estimate annual healthcare contacts for mood and anxiety disorders (specificity 92.2–93.4%; negative predictive value 96.0–96.9%), but not necessarily inferential to all diagnosed cases (sensitivity 64.7–71.5%; positive predictive value 47.0–53.2%) [35].

### 2.4. Neighbourhood Marginalization Measures

We considered the role of different measures of neighbourhood characteristics available through the (widely used but gender-neutral) Canadian Marginalization Index (CAN-Marg) [36], as well as sex-disaggregated data curated from census tables [37]. The CAN-Marg dataset quantifies four dimensions of neighbourhood socioeconomic status: material deprivation, residential instability, ethnic concentration, and population dependency [36]. These nationally consistent, multifaceted indices were generated from 2006 population census data and aggregated to the lowest level of standard census geographic units, i.e., dissemination areas (which typically have populations ~400–700 people) [38]. The four CAN-Marg dimensions describe a parsimonious set of neighbourhood characteristics associated theoretically and empirically with 18 health and behavioural outcomes, as retained following principal components factor analysis of an initial set of 42 indicators [39]. The only measure among this initial set that was distinguished by sex was the share of females in the total population, but it was not retained in the final index [39].

Since few studies have measured gender inequality at the neighbourhood level, especially as suited to smaller population centres, we sought to create regionally-optimized socioeconomic indicators disaggregated by sex. We therefore compiled sex-disaggregated provincial data by dissemination area for 11 single-item census measures [37], covering each of the CAN-Marg Index dimensions: (i) material deprivation: proportion of adults without a high school diploma, in a lone-parent family, or unemployed; (ii) residential instability: proportion living alone, non-youth, or not in a marital or common-law union; (iii) ethnic concentration: proportion who identified as a visible minority, proportion of five-year recent immigrants; and (iv) population dependency: proportion of seniors (aged 65 and over), labour force non-participation, and dependency ratio. We applied principal components analysis to optimize dimensionality by sex-specific neighbourhood-level socioenvironmental variables. Because research elsewhere suggests multifactor indices may be associated with greater uncertainty in identifying populations at higher risk of health inequalities in rural areas (i.e., compared with geographically smaller urban areas with equivalent population sizes) [6,40], we aimed to retain one single-item measure for each dimension (i.e., to reduce the above-mentioned set of 11 items to 4).

### 2.5. Statistical Analysis

We conducted a series of multivariate logistic regression analyses to examine associations between individuals’ demographic characteristics and neighbourhood environments with the risk of service contacts for mood and anxiety disorders, controlling for age category (i.e., the broad life cycle groupings representing childhood and youth, younger adulthood, middle-aged adulthood, and seniors), and stratified by sex at both the individual level and at the level of neighbourhood characterization. Residential neighbourhoods were ranked into quintiles, with higher quintiles representing more deprivation (i.e., Quintile 1 [Q1] = least marginalized areas, Quintile 5 [Q5] = most marginalized areas), a common approach for referencing population distributions of relative marginalization [4,36]. Adjusted odds ratios (ORs) and bootstrapped 95% confidence intervals (CIs) were estimated for each predictor. We limited analyses to individuals with non-missing data for all variables of interest. The study conformed to the REporting of studies Conducted using Observational Routinely-collected health Data (RECORD) protocol [41]. Population counts were rounded to a base of five to reinforce the confidential nature of the data.

### 2.6. Ethics Approval

This study was approved by the Research Ethics Board of the University of New Brunswick–Fredericton (REB #2017-076).

## 3. Results

### 3.1. Population

The population eligible for provincially-insured services totalled 775,225 persons in 2015/2016. After excluding those who did not have valid demographic or postal code information in the administrative data or who were younger than 1 year, the final analysis comprised 707,575 individuals (males: 348,030; females: 359,545) residing in 1374 neighbourhoods.

A disproportionately large percentage of the target population lived in neighbourhoods characterized with high material deprivation (37.6% residing in the highest areal deprivation quintile) and population dependency (25.0% in the highest areal dependency quintile) (Table 1). In comparison with nationally standardized measures, this smaller and largely rural province was described with relative ethnic homogeneity (2.5% residing in areas with high ethnic diversity) and residential stability (12.9% in areas of high residential instability).

One in ten (10.7%) residents experienced a mood or anxiety disorder entailing the use of medical or hospital services in the year. More females (14.0%) used mental health services compared with males (7.3%) (not shown). Adults of working age (25–64 years) were overrepresented among those having mental health service contacts compared with those in younger ages and with seniors (Table 1). The descriptive statistics suggested that users of mental health services were overrepresented compared with the total population among those in the most materially deprived neighbourhoods (38.9% vs. 37.6%) and the most residentially unstable areas (14.9% vs. 12.9%).

### 3.2. Gendered Neighbourhood Marginalization Metrics

The principal components analysis of neighbourhood-level census data revealed sex-specific divergences in the variation explained across most of the socioenvironmental indicators within the province (not shown). For each marginalization dimension, we retained for further analysis the measure affecting the largest variance for males and females: the proportion of adults without a high school diploma (material deprivation), the proportion living alone (residential instability), the dependency ratio (population dependence), and the proportion of visible minorities (ethnic concentration). Quintiles for the latter dimension were grouped, given New Brunswick’s relative ethnic homogeneity.

### 3.3. Risk of Service Contacts by Gender-Neutral Neighbourhood Characterization

Logistic regression results indicated a direct association between neighbourhood environments and mental health service contacts across two dimensions of (gender-neutral) marginalization: significantly higher odds among those residing in the most vs. least materially deprived neighbourhoods (OR: 1.09 [95% CI: 1.05–1.12]) and among those in the most vs. least residentially instable neighbourhoods (OR: 1.20 [95% CI: 1.16–1.25]) (Table 2, model 1). As expected, the odds of service use were significantly higher among females than males (OR: 2.07 [95% CI: 2.03–2.10]), and among adults of working age compared with children and youth or with seniors.

Following disaggregation of the analyses by individuals’ sex, the association between neighbourhood material deprivation with mental health service use among males (Table 2, model 2) and of neighbourhood population dependency among females (Table 2, model 3) attenuated to non-significance.

### 3.4. Risk of Service Contacts by Sex-Disaggregated Neighbourhood Characterization

Some differential associations were found in individuals’ risk of mental health service contacts according to whether the neighbourhood marginalization metrics were tallied among the male residential population, among females, or among both sexes combined. Individuals residing in neighbourhoods of the most female-specific material deprivation were significantly more likely to use mental health services compared with those in the least deprived neighbourhoods (OR: 1.06 [95% CI: 1.01–1.10]), an observation not found when considering neighbourhoods of male-specific deprivation (OR: 1.00 [95% CI: 0.96–1.04]) (Table 3, model 1). The observed patterns of higher service use among females and among those aged 25–44 were comparable to the results from the previous model using gender-neutral neighbourhood marginalization indices.

Following further disaggregation of the analyses by individuals’ sex, the association of living in the most female-specific materially deprived neighbourhoods with mental health service use was found to be significant among individual females (OR: 1.08 [95% CI: 1.02–1.14]) (Table 3, model 3), but not among males (OR: 1.02 [95% CI: 0.95–1.10]) (Table 3, model 2). Residence in neighbourhoods of highest male-specific deprivation did not exercise consistently significant influences on the outcome of interest among either individual males (OR: 0.95 [95% CI: 0.88–1.02]) (Table 3, model 2) or females (OR: 1.03 [95% CI: 0.98–1.09]) (Table 3, model 3).

## 4. Discussion

This study contributes to the (limited) literature seeking to identify which contextual features of gender inequality in residential environments might impact differently on women’s and men’s mental health service use. A growing body of evidence is providing insights on the role of neighbourhood environments as a social determinant of mental health; however, gender considerations have been largely overlooked in the measurement of neighbourhood-level characteristics [16]. Moreover, previous studies have tended to focus on residents of larger cities or on specific patient groups. The present study used linked individual-level and area-based datasets to provide a population-wide assessment of socioenvironmental equity in mental health outcomes through a gender lens, and this in the context of exclusively smaller urban and rural settlements. Neighbourhood stressors may potentially exacerbate women’s mental health [13]. We found, not unexpectedly, females were more likely than males to access healthcare services for mood and anxiety disorders (OR: 2.07 [95% CI: 2.03–2.10]), after adjusting for age and selected (commonly used but gender-neutral) metrics of neighbourhood socioenvironments. Our exploratory analysis further showed that, when considering only gender-neutral measures of neighbourhood marginalization, the adjusted risks of mental health service contacts were significantly higher among both males and females residing in neighbourhoods of higher residential instability; the sex-disaggregated risk patterns among residents of the most materially deprived neighbourhoods held for females but not for males. Even more complex patterns were observed when the geospatial measures were disaggregated by sex: residence in the most female-specific materially deprived neighbourhoods was independently associated with a higher risk of mental health service use among individual females (OR: 1.08 [95% CI: 1.02–1.14]) but not among males (OR: 1.02 [95% CI: 0.95–1.10]).

Our results were consistent with research elsewhere highlighting variations by sex in the impact of selected neighbourhood characteristics on mental health service contacts in this semi-rural population, suggesting that strategies aiming to improve care for mental disorders need to consider the distinct needs of women and men across socioenvironmental settings [31]. Given the lack of consensus in the literature on how to best describe the complexity of gender through multiple contextual dimensions [23], we operationalized sex-disaggregated indicators at both the individual and areal exposure levels. Women and men may experience distinctive vulnerabilities to different features of local environments, such as in perceptions of neighbourhood social cohesion and social roles normalizing time spent in the local area [17]. Gender-neutrality is increasingly recognized as associated with oversights and unanticipated negative impacts on women’s and men’s health in clinical research and practice; less evident is how measurable are the gendered norms and roles underlying observable differences in health outcomes between the sexes to better act on inequities at the population level [26]. More theory-driven and evidence-based research is needed on the dose–response relationships between gender, residential characteristics, and mental health.

Our findings were also consistent with increasing evidence that the relationships between neighbourhood characterization and mental health may differ from those related to physical health. While geospatial measures of population dependency have been widely related elsewhere to chronic physical health problems [36], we found few stable associations, likely reflecting age-specific patterns of mental service contacts. A recent Canadian study found no association between mental health outcomes and physical activity-friendliness of built environments [42]. A lack of statistical significance in certain hypothesized prediction patterns may be related to narrower between-neighbourhood sociodemographic differences within less-populated jurisdictions, e.g., small proportions of minority ethnic groups [25]. Further research should continue to explore which indicators of neighbourhood disadvantage are more closely associated with common mental health disorders across the lifespan and in different contexts of rurality.

### Limitations

Some limitations to this study are noted. Firstly, the datasets used here precluded the ability to disentangle biomedical “sex” differences from sociocultural “gender” continuum differences for understanding their relative contributions to the observed associations [43]. Second, given the cross-sectional nature of the analysis, causality cannot be implied; it is possible that persons with certain mental disorders may socially select into different types of neighbourhoods and housing, with the social causation processes potentially differing by gender given the higher mental health service utilization rates among women [44]. Third, information was lacking in the administrative datasets on person-level socioeconomic position, ethnolinguistic or cultural group, transportation barriers, and behavioural indicators that may mediate areal effects on mental health outcomes. Fourth, the numbers of those with mental health service contacts were likely underestimated, given the inability using the available datasets to capture those who used exclusively private or school-based services.

## 5. Conclusions

The rising prevalence of depression and other mood and anxiety disorders is of increasing concern to meet the demands for responsiveness of healthcare services while ensuring sustainability. It is widely recognized that actions to improve population health must extend outside the traditional boundaries of health systems. Many measurements have been developed to explore how selected neighbourhood characteristics might help reduce chronic stressors among women and men to help support decisions guiding appropriately targeted community-based interventions and resource allocations for mental health services [13,19]. However, there remains a lack of universal consensus on which metrics are most salient. In this study in a small urban and rural setting, we found evidence of sex-specific typologies of neighbourhood deprivation surrounding women’s risk of mental health service use compared to their male counterparts. As these findings need to be tested in other settings, our study aimed to raise cognition that gender needs to be an integral measurement at both the individual and contextual levels in research to inform neighbourhood designs to reduce the burden of mental disorders.

## Figures and Tables

**Table 1:** Percentage distribution of the population aged 1 year and over by selected person- and neighbourhood-level characteristics, according to healthcare utilization for mood and anxiety disorders, New Brunswick (Canada), 2015/2016.

Characteristic	Total Population Aged 1 Year and Over (*n* = 707,575)	Used Healthcare Services for a Mood or Anxiety Disorder (*n* = 76,045 [10.7%])
Sex		
Female	50.8%	66.4%
Male	49.2%	33.6%
Age group		
Children and youth (1–24 years)	25.0%	15.7%
Younger adults (25–44 years)	23.9%	31.0%
Middle-aged adults (45–64 years)	31.8%	36.5%
Seniors (65 years and over)	19.3%	16.8%
Neighbourhood material deprivation		
Quintile 1—least marginalized areas	12.9%	12.0%
Quintile 2	12.0%	11.6%
Quintile 3	16.3%	16.1%
Quintile 4	21.2%	21.4%
Quintile 5—most marginalized areas	37.6%	38.9%
Neighbourhood residential instability		
Quintile 1—least marginalized areas	12.3%	11.5%
Quintile 2	29.7%	28.0%
Quintile 3	27.2%	26.8%
Quintile 4	17.9%	18.8%
Quintile 5—most marginalized areas	12.9%	14.9%
Neighbourhood ethnic concentration		
Quintile 1—least marginalized areas	37.9%	36.9%
Quintile 2	30.4%	30.7%
Quintile 3	21.1%	21.5%
Quintile 4	8.1%	8.2%
Quintile 5—most marginalized areas	2.5%	2.7%
Neighbourhood population dependency		
Quintile 1—least marginalized areas	9.9%	9.7%
Quintile 2	13.0%	13.1%
Quintile 3	21.1%	20.4%
Quintile 4	31.0%	30.6%
Quintile 5—most marginalized areas	25.0%	26.2%
Total	**100%**	**100%**

Note: Neighbourhood characteristics based on gender-neutral composite indices of marginalization. Source: New Brunswick administrative health datasets for 2015/2016 linked to the 2006 CAN-Marg dataset.

**Table 2:** Adjusted odds ratios (and 95% confidence intervals) for the risk of healthcare utilization for mood and anxiety disorders—regression models disaggregated by sex at the individual level.

Characteristic	(1) Total (*n* = 707,575)			(2) Males (*n* = 348,030)			(3) Females (*n* = 359,545)		
	OR	95% CI	*p*-Value	OR	95% CI	*p*-Value	OR	95% CI	*p*-Value
Sex (ref: Male)									
Female	2.07 *	2.03–2.10	0.00	-			-		
Age group (ref: 25–44 years)									
1–24 years	0.45 *	0.44–0.46	0.00	0.49 *	0.47–0.50	0.00	0.43 *	0.42–0.44	0.00
45–64 years	0.87 *	0.86–0.89	0.00	0.94 *	0.91–0.97	0.00	0.84 *	0.82–0.86	0.00
65+ years	0.61 *	0.60–0.62	0.00	0.70 *	0.68–0.72	0.00	0.57 *	0.55–0.58	0.00
Neighbourhood material deprivation (ref: Q1–least marginalized areas)									
Q2	1.02	0.99–1.05	0.25	0.99	0.95–1.04	0.75	1.03	0.98–1.08	0.20
Q3	1.03	0.99–1.06	0.11	0.98	0.93–1.03	0.51	1.05 *	1.01–1.09	0.02
Q4	1.04 *	1.01–1.08	0.01	0.98	0.93–1.03	0.33	1.08 *	1.04–1.13	0.00
Q5–most marginalized	1.09 *	1.05–1.12	0.00	1.00	0.96–1.05	0.91	1.13 *	1.09–1.17	0.00
Neighbourhood residential instability (ref: Q1–least marginalized areas)									
Q2	0.99	0.96–1.02	0.53	0.98	0.94–1.03	0.44	0.99	0.96–1.03	0.75
Q3	1.03 *	1.01–1.06	0.02	1.02	0.98–1.07	0.31	1.04	1.00–1.08	0.08
Q4	1.10 *	1.07–1.14	0.00	1.12 *	1.07–1.17	0.00	1.09 *	1.06–1.13	0.00
Q5–most marginalized	1.20 *	1.16–1.25	0.00	1.27 *	1.21–1.33	0.00	1.17 *	1.12–1.22	0.00
Neighbourhood ethnic concentration (ref: Q1–least marginalized areas)									
Q2	1.04 *	1.02–1.06	0.00	1.04 *	1.01–1.08	0.01	1.04 *	1.02–1.07	0.00
Q3	1.04 *	1.01–1.06	0.00	1.04	0.99–1.08	0.11	1.03 *	1.01–1.06	0.02
Q4	0.99	0.95–1.02	0.48	1.00	0.93–1.06	0.88	0.98	0.94–1.02	0.40
Q5–most marginalized	1.01	0.96–1.07	0.63	0.94	0.85–1.04	0.21	1.05	0.98–1.13	0.18
Neighbourhood population dependency (ref: Q1–least marginalized areas)									
Q2	1.04 *	1.00–1.07	0.04	1.02	0.95–1.09	0.54	1.05	1.00–1.10	0.07
Q3	0.99	0.96–1.02	0.48	1.01	0.96–1.07	0.75	0.98	0.93–1.03	0.37
Q4	1.00	0.97–1.03	0.97	1.03	0.97–1.09	0.31	0.99	0.94–1.03	0.58
Q5–most marginalized	1.04 *	1.01–1.07	0.02	1.09 *	1.02–1.16	0.01	1.02	0.97–1.06	0.45

* *p* < 0.05; ref = reference category; OR = odds ratio; CI = confidence interval; Q = quintile. Neighbourhood characteristics based on gender-neutral composite indices of marginalization. Source: New Brunswick administrative health datasets for 2015/2016 linked to the 2006 CAN-Marg dataset.

**Table 3:** Adjusted odds ratios (and 95% confidence intervals) for the risk of healthcare utilization for mood and anxiety disorders—regression models disaggregated by sex at the individual and neighbourhood levels.

Characteristic	(1) Total (*n* = 707,575)			(2) Males (*n* = 348,030)			(3) Females (*n* = 359,545)		
	OR	95% CI	*p*-Value	OR	95% CI	*p*-Value	OR	95% CI	*p*-Value
Sex (ref: Male)									
Female	2.07 *	2.03–2.10	0.00	-			-		
Age group (ref: 25–44 years)									
1–24 years	0.45 *	0.44–0.46	0.00	0.49 *	0.46–0.51	0.00	0.43 *	0.42–0.45	0.00
45–64 years	0.87 *	0.85–0.88	0.00	0.94 *	0.90–0.97	0.00	0.83 *	0.81–0.86	0.00
65+ years	0.61 *	0.59–0.62	0.00	0.70 *	0.67–0.73	0.00	0.57 *	0.55–0.58	0.00
Neighbourhood material deprivation: areal measure among both genders combined (ref: Q1–least marginalized areas)									
Q2	1.06 *	1.03–1.10	0.00	1.08 *	1.02–1.13	0.01	1.06 *	1.02–1.10	0.01
Q3	1.04 *	1.00–1.07	0.03	1.05	0.99–1.11	0.13	1.03	0.98–1.09	0.27
Q4	1.03	0.99–1.08	0.15	1.05	0.97–1.15	0.24	1.02	0.96–1.09	0.54
Q5–most marginalized	1.05	0.99–1.11	0.10	1.09	0.99–1.21	0.08	1.03	0.95–1.11	0.51
Neighbourhood material deprivation: areal measure among males (ref: Q1–least marginalized areas)									
Q2	1.00	0.97–1.03	0.92	1.01	0.96–1.05	0.81	0.99	0.96–1.03	0.75
Q3	1.03	1.00–1.05	0.07	1.02	0.96–1.08	0.52	1.03	0.98–1.08	0.27
Q4	1.05 *	1.01–1.08	0.01	1.00	0.93–1.07	0.92	1.07 *	1.02–1.13	0.01
Q5–most marginalized	1.00	0.96–1.04	0.90	0.95	0.88–1.02	0.15	1.03	0.98–1.09	0.21
Neighbourhood material deprivation: areal measure among females (ref: Q1–least marginalized areas)									
Q2	1.02	0.99–1.05	0.16	0.98	0.93–1.03	0.42	1.05 *	1.00–1.09	0.03
Q3	1.05 *	1.02–1.09	0.00	1.03	0.97–1.09	0.40	1.07 *	1.02–1.13	0.01
Q4	1.05 *	1.01–1.09	0.02	1.00	0.93–1.07	0.97	1.08 *	1.03–1.13	0.00
Q5–most marginalized	1.06 *	1.01–1.10	0.01	1.02	0.95–1.10	0.56	1.08 *	1.02–1.14	0.01
Neighbourhood residential instability: areal measure among both genders combined (ref: Q1–least marginalized areas)									
Q2	0.98	0.96–1.01	0.24	0.97	0.93–1.02	0.23	0.99	0.96–1.03	0.69
Q3	0.97	0.93–1.00	0.05	0.95 *	0.90–1.00	0.04	0.98	0.94–1.01	0.23
Q4	0.94 *	0.90–0.99	0.01	0.92 *	0.86–0.99	0.03	0.95	0.89–1.02	0.13
Q5–most marginalized	1.01	0.95–1.07	0.70	1.05	0.94–1.16	0.39	0.99	0.92–1.07	0.89
Neighbourhood residential instability: areal measure among males (ref: Q1–least marginalized areas)									
Q2	1.01	0.99–1.04	0.38	1.01	0.96–1.05	0.80	1.01	0.98–1.05	0.40
Q3	0.99	0.96–1.01	0.31	0.95 *	0.91–0.99	0.02	1.01	0.97–1.04	0.75
Q4	1.04 *	1.00–1.08	0.04	1.02	0.97–1.08	0.47	1.05 *	1.01–1.09	0.01
Q5–most marginalized	1.09 *	1.05–1.13	0.00	1.06	0.99–1.12	0.08	1.11 *	1.06–1.15	0.00
Neighbourhood residential instability: areal measure among females (ref: Q1–least marginalized areas)									
Q2	1.02	0.99–1.05	0.11	1.05	1.00–1.10	0.05	1.01	0.98–1.04	0.46
Q3	1.05 *	1.01–1.08	0.01	1.04	0.98–1.10	0.16	1.05 *	1.02–1.09	0.00
Q4	1.07 *	1.03–1.12	0.00	1.09 *	1.02–1.16	0.01	1.06 *	1.02–1.11	0.01
Q5–most marginalized	1.09 *	1.04–1.15	0.00	1.13 *	1.05–1.22	0.00	1.07 *	1.01–1.14	0.03
Neighbourhood ethnic concentration: areal measure among both genders combined (ref: Q1–least marginalized areas)									
Q2–Q4	1.02 *	1.00–1.05	0.04	1.01	0.96–1.06	0.80	1.04 *	1.00–1.07	0.03
Q5–most marginalized	0.98	0.94–1.02	0.29	0.97	0.90–1.04	0.37	0.98	0.93–1.04	0.55
Neighbourhood ethnic concentration: areal measure among males (ref: Q1–least marginalized areas)									
Q2–Q4	1.00	0.96–1.03	0.80	0.99	0.94–1.05	0.81	1.00	0.95–1.04	0.85
Q5–most marginalized	0.99	0.96–1.02	0.53	0.98	0.93–1.03	0.39	1.00	0.96–1.03	0.81
Neighbourhood ethnic concentration: areal measure among females (ref: Q1–least marginalized areas)									
Q2–Q4	1.05 *	1.02–1.09	0.00	1.01	0.95–1.07	0.68	1.07 *	1.04–1.11	0.00
Q5–most marginalized	0.99	0.96–1.03	0.71	0.98	0.93–1.04	0.56	1.00	0.96–1.03	0.92
Neighbourhood population dependency: areal measure among both genders combined (ref: Q1–least marginalized areas)									
Q2	1.02	0.99–1.06	0.19	0.99	0.94–1.04	0.74	1.04	0.99–1.09	0.11
Q3	1.00	0.96–1.04	0.87	0.98	0.91–1.05	0.51	1.01	0.95–1.06	0.80
Q4	1.08 *	1.04–1.12	0.00	1.05	0.99–1.12	0.12	1.09 *	1.03–1.15	0.00
Q5–most marginalized	1.07 *	1.02–1.12	0.01	1.01	0.94–1.10	0.73	1.09 *	1.03–1.16	0.01
Neighbourhood population dependency: areal measure among males (ref: Q1–least marginalized areas)									
Q2	0.96 *	0.94–0.99	0.00	0.96	0.91–1.01	0.08	0.97 *	0.94–1.00	0.04
Q3	0.96 *	0.93–0.99	0.01	0.96	0.91–1.02	0.18	0.96 *	0.92–1.00	0.04
Q4	0.96 *	0.93–0.99	0.01	0.93 *	0.88–0.99	0.02	0.98	0.94–1.02	0.29
Q5–most marginalized	0.97 *	0.93–1.00	0.04	0.93 *	0.87–1.00	0.04	0.99	0.94–1.03	0.54
Neighbourhood population dependency: areal measure among females (ref: Q1–least marginalized areas)									
Q2	1.02	0.99–1.05	0.12	1.05	1.00–1.10	0.06	1.01	0.97–1.04	0.67
Q3	0.98	0.95–1.01	0.14	1.02	0.96–1.08	0.61	0.96	0.92–1.00	0.07
Q4	0.95 *	0.93–0.99	0.00	1.02	0.96–1.08	0.50	0.92 *	0.88–0.96	0.00
Q5–most marginalized	0.96 *	0.93–1.00	0.04	1.04	0.98–1.11	0.20	0.92 *	0.87–0.98	0.00

* *p* < 0.05; ref = reference category; OR = odds ratio; CI = confidence interval; Q = quintile. Neighbourhood characteristics based on gender-specific single-item measures of marginalization and province-specific distributions. Source: New Brunswick administrative health datasets for 2015/2016 linked to area-based 2006 census data from authors’ calculations.

## Data Availability

The data that support the findings of this study are available from the New Brunswick Institute for Research, Data and Training (NB-IRDT) but restrictions apply to the accessibility of these data, which were used under license for the current study. Researchers wishing to request access to the datasets may submit an application to the NB-IRDT (www.unb.ca/nbirdt).
